# Influence of Immersion Level and Game Type on Virtual Reality Interventions for Upper Limb Function After Stroke: Protocol for a Systematic Review and Meta-Analysis

**DOI:** 10.2196/95135

**Published:** 2026-10-06

**Authors:** Dayenne Jeneffer Souza da Silva, Vitor Leandro da Cunha, Luanna Barbara de Araújo Farias, Lara Cecília de Araújo Carlos, Candice Simões Pimenta de Medeiros, Edgar Ramos Vieira, Fabrícia Azevedo da Costa Cavalcanti

**Affiliations:** 1 Universidade Federal do Rio Grande do Norte Natal, Rio Grande do Norte Brazil; 2 Ceará School of Public Health Health Residency Program Ceará, Fortaleza Brazil; 3 Florida International University Florida, FL United States

**Keywords:** virtual reality, video games, serious games, commercial games, upper extremity, stroke, exergaming, digital health

## Abstract

**Background:**

Upper limb impairment is a common consequence of stroke, affecting independence and quality of life. Virtual reality (VR) interventions, including serious games designed for therapeutic purposes and commercial games developed primarily for entertainment, have been increasingly used for poststroke upper limb rehabilitation. Evidence comparing the effectiveness of these game types on functional outcomes remains unclear.

**Objective:**

This systematic review protocol aims to determine whether serious and commercial VR games differentially impact upper limb function, measured by the Action Research Arm Test, in adults after stroke. Secondary outcomes will include upper limb motor function assessed using the Fugl-Meyer Assessment-Upper Extremity, Box and Block Test, Motor Activity Log, Jebsen-Taylor Hand Function Test, and Wolf Motor Function Test; functional independence assessed using the Functional Independence Measure and Barthel index; and adherence indicators, including dropout rates and session completion. Secondary objectives include evaluating pooled VR effects vs conventional therapy (CT) and exploring potential moderators, such as VR immersion level and stroke chronicity.

**Methods:**

Parallel randomized controlled trials involving adults (aged ≥18 years) with ischemic or hemorrhagic stroke in the acute, subacute, or chronic phases will be included. Interventions using serious or commercial VR games (nonimmersive, semi-immersive, or fully immersive) delivered alone or combined with CT will be considered. Studies using nongamified VR, digital technologies without VR, or augmented reality not integrated into VR will be excluded. Eligible comparators include conventional physiotherapy, occupational therapy, usual care, educational interventions, or no therapy. This protocol was developed in accordance with the PRISMA-P (Preferred Reporting Items for Systematic Reviews and Meta-Analyses-Protocols) guidelines. Systematic searches will be conducted in the PubMed (MEDLINE), Web of Science, Embase, Scopus, Virtual Health Library, ScienceDirect, Physiotherapy Evidence Database, Cochrane Central Register of Controlled Trials, ClinicalTrials.gov, Brazilian Clinical Trials Registry, World Health Organization (WHO) International Clinical Trial Registry Platform, and ISRCTN Registry databases from inception to February 2026. Data extraction will be performed independently by 2 reviewers, and risk of bias will be assessed using the Cochrane risk of bias 2.0 tool. Meta-analyses will be conducted when clinical and methodological homogeneity permits. A random-effects model is planned; however, the final choice of model will be based on the level of heterogeneity observed at the time of analysis. Subgroup analyses will explore stroke phase, immersion level, intervention dosage, and concomitant therapy.

**Results:**

Data collection is planned from inception to February 2026. Study screening and data extraction are ongoing, with synthesis and meta-analysis expected by November 2026 and final publication anticipated in winter 2026.

**Conclusions:**

This systematic review will clarify whether different VR game formats produce distinct effects on upper limb rehabilitation after stroke, providing evidence to inform the design and implementation of VR-based neurorehabilitation interventions.

**Trial Registration:**

PROSPERO CRD42024595266; https://www.crd.york.ac.uk/PROSPERO/view/CRD42024595266

**International Registered Report Identifier (IRRID):**

DERR1-10.2196/95135

## Introduction

Stroke is one of the leading causes of mortality and long-term disability worldwide, with persistent upper limb impairments affecting a large proportion of survivors [1,2]. These deficits compromise goal-directed movements, such as reaching and object interaction, which are essential for independence in activities of daily living [2,3]. Virtual reality (VR) has emerged as a promising tool in poststroke rehabilitation due to its ability to provide repetitive, task-oriented training within controlled and engaging environments [4-7]. Previous studies suggest that VR-based interventions may enhance upper limb recovery, particularly when combined with conventional therapy (CT) [2,3,6]. However, substantial heterogeneity exists across VR interventions, including differences in technological platforms, levels of immersion, and characteristics of the virtual tasks [2,3,7].

One critical and insufficiently explored aspect of VR-based rehabilitation concerns the nature of the virtual tasks themselves. Serious games are intentionally designed to target therapeutic goals and often incorporate structured motor demands aligned with rehabilitation principles. In contrast, commercial games are primarily developed for entertainment, although they may still promote active movement and engagement [8-10]. While both formats are widely adopted in rehabilitation settings, their therapeutic equivalence remains uncertain. Additional complexity arises from differences in immersion, as VR systems span a continuum from nonimmersive to fully immersive environments, which may differentially influence engagement, multisensory integration, and motor performance. However, immersion level and game design frequently coexist in experimental protocols, making it difficult to determine whether functional outcomes are driven by technological features, task structure, or their interaction [11-14].

Most studies evaluate VR as a unified intervention without differentiating the therapeutic contribution of specific game formats. Although there is a growing assumption that serious games may lead to greater functional gains due to their emphasis on task-specific training, this hypothesis remains largely speculative and has not been systematically investigated [3,15-18]. From a motor learning perspective, repetition of task-specific movements that resemble real-world activities may enhance the transfer of skills acquired in virtual environments to functional performance outside the virtual context. In contrast, commercial games, although not specifically designed for rehabilitation, may enhance engagement, motivation, and adherence, which are key factors influencing training intensity and overall dosage. These distinct mechanisms suggest that different types of games may contribute to rehabilitation outcomes through separate pathways. Therefore, the relative contributions of task-specific training and engagement-driven mechanisms remain unclear, and the available evidence is limited and heterogeneous [18-21].

Given the central role of functional upper limb recovery in poststroke rehabilitation, clarifying whether serious and commercial games produce distinct effects on functional performance is clinically relevant. Clarifying this point is essential for advancing the evidence-based implementation of VR in neurorehabilitation [21-25]. Therefore, this systematic review will evaluate the effects of VR-based interventions on upper limb function in adults after stroke, with the Action Research Arm Test (ARAT) defined as the primary outcome. Eligible interventions may involve VR applied alone or combined with CT or occupational therapy (OT). Comparator groups may include CT, OT, usual care, educational interventions, or no intervention. In addition, subgroup analyses will explore whether different VR game formats, categorized as serious or commercial, are associated with variations in treatment effects. Potential sources of heterogeneity, including immersion level and stroke chronicity, will also be investigated.

## Methods

This systematic review protocol will be conducted in accordance with the PRISMA-P (Preferred Reporting Items for Systematic Reviews and Meta-Analyses-Protocols) guidelines [25]. This protocol has been registered with the International Prospective Register of Systematic Reviews (PROSPERO; CRD42024595266).

### Eligibility Criteria

The eligibility criteria will be established according to the participants, interventions, comparisons, outcomes, and study design (PICOS) strategy.

### Participants

Studies including adults aged ≥18 years with a confirmed diagnosis of ischemic or hemorrhagic stroke in the acute (<1 month), subacute (1-6 months), or chronic phase (>6 months) will be eligible. The time since stroke onset must be clearly reported. Participants of any sex presenting with mild-to-severe upper limb motor impairment will be included [26]. The level of impairment will be defined according to the clinical measures and classification reported in each study. Studies conducted in hospital settings, outpatient clinics, home-based rehabilitation, laboratory environments, or through telerehabilitation will be included. Studies involving participants with other neurological or systemic conditions that may independently affect motor performance will be excluded.

### Intervention Characteristics

Studies implementing VR-based interventions using serious or commercial games for upper limb rehabilitation in individuals after stroke will be included. Interventions may involve nonimmersive VR (NIVR), semi-immersive VR (SIVR), or fully immersive VR (FIVR) systems. The level of immersion will be classified according to the hardware configuration and display characteristics described in each study. Augmented reality systems will be included only when integrated into VR-based platforms.

Eligible systems may include console-based or motion-capture technologies, such as Nintendo Wii, Xbox Kinect, or PlayStation platforms, as well as head-mounted display devices used in fully immersive environments, including Oculus Quest, Apple Vision Pro, or HTC Vive [21,27,28]. Studies using nongamified virtual environments, digital technologies without VR, or augmented reality systems not integrated into VR will be excluded. Studies in which the type of game cannot be classified as serious or commercial according to the predefined criteria will also be excluded.

Serious games will be defined as games designed with explicit therapeutic intent beyond entertainment, incorporating structured motor demands aligned with health-related objectives. Commercial games will be defined as games developed primarily for entertainment and subsequently used in rehabilitation [9]. Game classification will be performed by the reviewers according to these predefined conceptual criteria based on the information provided in each study. When not explicitly stated, classification will follow the predefined criteria of this review. Classification will be performed independently by 2 reviewers, with disagreements resolved by consensus or consultation with a third reviewer.

Studies in which VR is applied alone or combined with CT or OT in the experimental group will be included. The influence of concomitant therapies will be addressed during data synthesis. The primary comparison will consider VR-based interventions applied alone or combined with CT or OT, regardless of game type, vs control conditions, including CT, OT, usual care, or no intervention. When data are available, subgroup analyses will be conducted to explore whether different VR game formats, categorized as serious or commercial, are associated with variations in treatment effects. Additional subgroup analyses may include comparisons based on the level of immersion, when applicable.

### Comparisons

The primary comparison will evaluate the effects of VR interventions on upper limb function, measured by the ARAT, compared with control conditions. Control groups may include CT, OT, educational interventions, usual care, or no therapy. Interventions will be categorized as serious or commercial VR games, and effect estimates will be examined separately according to this classification.

Secondary comparisons will include pooled VR games vs CT or OT and VR delivered alone vs VR combined with concomitant CT or OT. When applicable, analyses will distinguish between these conditions to account for the potential influence of combined interventions. Studies using nongamified virtual environments, digital technologies without VR, or augmented reality systems not integrated into VR will be excluded. Studies in which the type of game cannot be classified as serious or commercial according to the predefined criteria will also be excluded.

### Outcomes

The primary outcome will be functional upper limb performance assessed using the ARAT. The ARAT was selected due to its strong clinical validity, responsiveness to change, and frequent use as a primary outcome measure in stroke rehabilitation trials, allowing greater comparability across studies. We will consider thresholds of 12 points for the dominant side and 17 points for the nondominant side, based on previously reported minimal clinically important difference (MCID) values, to identify whether observed changes are likely to represent clinically meaningful improvements. These thresholds will be used to support the interpretation of ARAT score changes and will be considered alongside effect size and statistical significance to contextualize clinical relevance [29,30].

The following secondary outcomes will be considered. Upper limb motor function will be assessed using the Fugl-Meyer Assessment-Upper Extremity (FMA-UE), Box and Block Test (BBT), Motor Activity Log (MAL), Jebsen-Taylor Hand Function Test (JTHFT), and Wolf Motor Function Test (WMFT). The MCID values reported in the literature will be considered for interpretation of these outcomes. For the FMA-UE, MCID values ranging from 4 points will be considered. For the WMFT time score, an MCID of 19 seconds on the affected dominant side will be considered. For the MAL, an MCID of at least 1 point will be considered. For the BBT and JTHFT, the MCID will be interpreted according to the values reported in the included studies and the characteristics of the evaluated population, as no universally accepted threshold for individuals after stroke has been established [30-33].

Functional independence will be assessed using the Functional Independence Measure (FIM) and Barthel index (BI). The MCID values reported in the literature will be considered for interpretation of these outcomes. For the BI, MCID values ranging from 4 to 5 points will be considered for individuals after stroke. For the FIM, a 22-point improvement in the total score will be considered clinically meaningful for patients who had a stroke [31,34].

Treatment adherence indicators will be assessed using dropout rates and session completion [35].

Exploratory outcomes will encompass the following. Kinematic measures will include joint angles, movement smoothness, trajectory parameters, or other biomechanical indicators of paretic upper limb performance obtained through goniometry or motion analysis systems [36]. Motivation and engagement will be assessed using the Intrinsic Motivation Inventory (IMI) or other validated self-reported measures described in the included studies. For the IMI, MCID values reported in the included studies will be considered when available. Adverse events will include cybersickness or other self-reported incidents.

### Study Design

Parallel randomized controlled trials evaluating serious or commercial VR games for upper limb rehabilitation after stroke will be included. Eligible comparators include CT, OT, usual care, educational interventions, or no intervention. Crossover trials and nonrandomized study designs will be excluded. Studies in which robot-assisted therapy, neuromodulation, or mirror therapy represent the primary component of the experimental intervention, as defined by the main therapeutic focus described by the authors, will be excluded to isolate the effects of VR-based interventions. Studies that do not provide sufficient information to identify or classify the VR intervention, such as those lacking a game description, platform, or therapeutic purpose, will also be excluded.

### Search Strategy

A systematic search will be conducted across the following electronic databases from inception to February 2026: PubMed (MEDLINE), Web of Science, Embase (Elsevier), Scopus (Elsevier), Virtual Health Library, ScienceDirect (Elsevier), Physiotherapy Evidence Database (PEDro), and Cochrane Central Register of Controlled Trials. Additional searches will be conducted on ClinicalTrials.gov [37], Brazilian Clinical Trials Registry [38], World Health Organization (WHO) International Clinical Trial Registry Platform, and ISRCTN Registry to identify ongoing or unpublished trials. Search strategies will combine controlled vocabulary terms, such as MeSH and Emtree, and free-text terms related to stroke, upper extremity, VR, and exergaming, using Boolean operators and database-specific adaptations. The full search strategies for each database are presented in Multimedia Appendix 1.

An extended search will include screening the reference lists of included studies and relevant reviews, as well as searching gray literature sources when applicable. Authors and field experts may be contacted when necessary to obtain missing or unpublished data. No language restrictions will be applied. A PubMed alert will be activated to identify newly published studies meeting the eligibility criteria until the final stage of manuscript preparation.

### Study Selection

Two authors (DJSDS and VLdC) will independently screen retrieved records. Records will be imported and managed using Rayyan (Rayyan Intelligent Systematic Review tool) to facilitate title and abstract screening and the exclusion of irrelevant articles [39]. Duplicate studies will be removed prior to eligibility assessment. Full texts of potentially eligible studies will then be retrieved and independently assessed by the same reviewers. Disagreements will be resolved through discussion, and a third reviewer (LBdAF) will be consulted when consensus cannot be reached. Reasons for exclusion at the full-text stage will be documented in detail. The study selection process will be reported using a PRISMA (Preferred Reporting Items for Systematic Reviews and Meta-Analyses) flow diagram.

### Data Extraction

A standardized data extraction form will be developed through consensus among all authors to systematically capture study characteristics essential for the primary analysis. This will include publication details (year and authors), methodological features (study design, duration, and randomization methods), and population characteristics (age, sex, stroke type and chronicity, upper limb impairment severity, and affected side). Intervention data relevant to the serious vs commercial game comparison will specifically include (1) game classification according to predefined criteria; (2) immersion level categorized as nonimmersive, semi-immersive, or fully immersive VR; (3) hardware specifications, such as console-based or head-mounted display systems; and (4) dosage parameters, including total training hours, number of sessions, session duration, and weekly frequency. Information on concomitant therapies will also be extracted.

Outcome data will include preintervention; postintervention; and, when available, follow-up values for both intervention and comparator groups. For continuous outcomes, means, SDs, and sample sizes will be extracted. When change scores are reported, these will be prioritized; otherwise, postintervention values will be used. If necessary, data reported as medians and IQRs will be converted using established methods.

Primary outcome data will include the ARAT. Secondary outcomes will include the FMA-UE, BBT, MAL, JTHFT, WMFT, FIM, BI, and treatment completion indicators, such as dropout rates and session adherence. Additional data will include information on attrition rates, intention-to-treat analysis, and funding sources, when reported. Two reviewers (DJSDS and VLdC) will independently extract data from included studies. Discrepancies will be resolved through discussion, and a third reviewer (FAdCC) will be consulted if consensus cannot be reached.

### Data Analysis and Synthesis

Quantitative synthesis will be conducted using Review Manager (RevMan; version 5.4; Cochrane) when ≥5 studies demonstrate sufficient clinical or methodological homogeneity [40]. A random-effects model is planned due to anticipated variability in participant characteristics, stroke phase, intervention protocols, and immersion levels; however, the final choice of model will be confirmed based on the level of heterogeneity observed at the time of analysis. The primary analysis will estimate the effects of serious and commercial VR games on upper limb function measured by the ARAT. Direct head-to-head randomized controlled trials will be synthesized separately when available. When direct comparisons are insufficient, indirect comparisons between serious and commercial games will be explored through predefined game-type subgroup analyses within VR vs control trials.

Subgroup analyses will be considered exploratory and will only be conducted when a sufficient number of studies is available to support meaningful comparisons. When data are limited, subgroup analyses will not be performed, and findings will be described narratively. When multiple publications from the same trial are identified, they will be grouped and treated as a single study. In cases of duplicate reports, the primary publication containing the most complete methodological and outcome data will be used. Additional reports providing complementary information, such as different outcomes or follow-up assessments, will be used to supplement data extraction while avoiding double counting of participants. When available, follow-up outcome data will be extracted and included in the analysis when studies report comparable time points; otherwise, these data will be summarized narratively.

Meta-analyses will be performed when at least 5 studies reporting sufficiently homogeneous outcomes are available for a given comparison. Planned analyses will include pooled VR interventions vs control conditions, as well as VR delivered alone vs VR combined with concomitant CT or OT. Separate meta-analyses will be conducted for each outcome measure whenever appropriate. For outcomes assessed using the same measurement instrument, pooled effects will be calculated using mean differences with 95% CIs. Standardized mean differences will be used when different instruments are used to assess the same underlying construct. Binary outcomes will be summarized using risk ratios with 95% CIs. When fewer than 5 studies are available for a specific outcome, or when substantial clinical or methodological heterogeneity precludes quantitative synthesis, findings will be presented through a structured narrative synthesis.

### Subgroup Analyses

Prespecified subgroup analyses will be conducted when at least 5 studies per comparison are available to explore potential sources of heterogeneity. Subgroup differences will be examined using the interaction test implemented in RevMan [40]. Subgroup analyses will include stroke phase, among other variables. When studies include participants across multiple stroke phases or report insufficient information to allow clear classification, they will be categorized as mixed or unclear phase. These studies will be included in the overall analysis but excluded from subgroup analyses based on stroke phase. Results will be interpreted cautiously due to the exploratory nature of subgroup analyses, the risk of reduced statistical power, and the observational nature of between-study comparisons.

The primary subgroup analysis, aligned with the research question, will compare game types categorized as serious or commercial VR games according to the operational definitions established in the Intervention Characteristics section. Secondary subgroup analyses will include stroke phase, defined as acute (<1 month after onset), subacute (1-6 months), or chronic (>6 months); immersion level, categorized as NIVR, SIVR, or FIVR systems; and intervention dosage, dichotomized by total training hours as less than 20 hours vs 20 hours or more. This threshold was defined based on typical intervention protocols reported in the literature, in which sessions commonly last 20 to 30 minutes and are delivered over multiple weeks, resulting in cumulative exposure within this range [13,17,20,23]. This categorization will be used as an exploratory approach to investigate potential dose-response effects.

Exploratory subgroup analyses, when at least 10 studies are available overall, will examine concomitant therapy, defined as VR delivered alone vs VR combined with CT, as well as age group, categorized as less than 65 years vs 65 years or more, and sex distribution, categorized according to majority female vs majority male samples. Subgroup findings will be interpreted with consideration of potential ecological bias and the inherent limitations of indirect comparisons across trials.

### Data Heterogeneity

Heterogeneity among included studies will be assessed qualitatively by comparing key study characteristics, including population profiles, intervention protocols, outcome measures, and analytical methods, and quantitatively using the *I*² statistic, Cochran *Q* test with a *P*<.10 threshold, and the τ² estimate of between-study variance. The *I*² statistic estimates the proportion of total variability attributable to between-study differences rather than sampling error, with values >50% interpreted as indicative of substantial heterogeneity [41].

A random-effects meta-analysis model will be applied because of anticipated clinical and methodological diversity across studies, providing more conservative pooled effect estimates. When substantial heterogeneity is identified, defined as *I*² >50% or *Q* test *P*<.10, predefined subgroup analyses will be conducted to explore potential sources of variability. If heterogeneity remains unexplained following subgroup exploration, findings will be interpreted cautiously, and a structured narrative synthesis supported by summary tables may be considered in place of quantitative pooling [36].

### Risk of Bias Assessment

Risk of bias will be evaluated using the Cochrane risk of bias (RoB) 2.0 tool by 2 independent reviewers (FAdCC and CSPdM). This assessment will guide the interpretation of the results and inform the meta-analysis. Five domains will be assessed: (1) bias arising from the randomization process, (2) bias due to deviations from intended interventions, (3) bias due to missing outcome data, (4) bias in outcome measurement, and (5) bias in selection of the reported result. Each domain will be judged as having low risk of bias, some concerns, or high risk of bias according to the signaling questions and decision algorithms described in the Cochrane Handbook. An overall risk of bias judgment will be assigned to each study following Cochrane guidance.

Studies judged as having high risk of bias will be included in the primary meta-analyses but excluded from sensitivity analyses to assess the robustness of pooled effect estimates. Disagreements between reviewers will be resolved through discussion, with consultation with a third reviewer (LCdAC) if necessary. Results will be presented in tabular format as risk of bias summaries and in graphical format using traffic light plots.

### Evidence Quality

The certainty of evidence will be assessed using the GRADE (Grading of Recommendations Assessment, Development and Evaluation) approach by 2 independent reviewers (FAdCC and CSPdM). The certainty of evidence for each outcome will be rated as high, moderate, low, or very low based on 5 domains: risk of bias, consistency of effect, imprecision, indirectness, and publication bias [42,43]. To complement the methodological characterization of the studies, the PEDro scale will also be applied. The PEDro scale comprises 11 items, of which 10 contribute to the total score, as the first item evaluates external validity and is not included in the final scoring. The scored criteria assess random allocation; concealed allocation; baseline comparability; blinding of participants, therapists, and assessors; adequacy of follow-up; intention-to-treat analysis; between-group comparisons; and reporting of point estimates and variability measures [44].

Rather than applying strict cutoff values, PEDro scores will be interpreted along a continuum, acknowledging the inherent challenges of blinding in rehabilitation trials. The PEDro assessment will be used solely to characterize the methodological profile of included studies and will not replace the domain-based risk of bias evaluation conducted using the RoB 2 tool. Two independent reviewers (FdACC and CSPdM) will perform the assessment, with disagreements resolved through discussion and consultation with a third reviewer (LCdAC) when necessary. PEDro scores will be reported descriptively.

To improve the conceptual and methodological transparency of the review, a visual scientific framework was developed to summarize the theoretical rationale, study design, eligibility criteria, screening process, outcome selection, and planned interpretation of findings. This framework illustrates the proposed relationships between VR game type, immersion level, upper limb functional outcomes, and their potential implications for clinical decision-making in neurofunctional rehabilitation after stroke (Figure 1).

**Figure 1:**
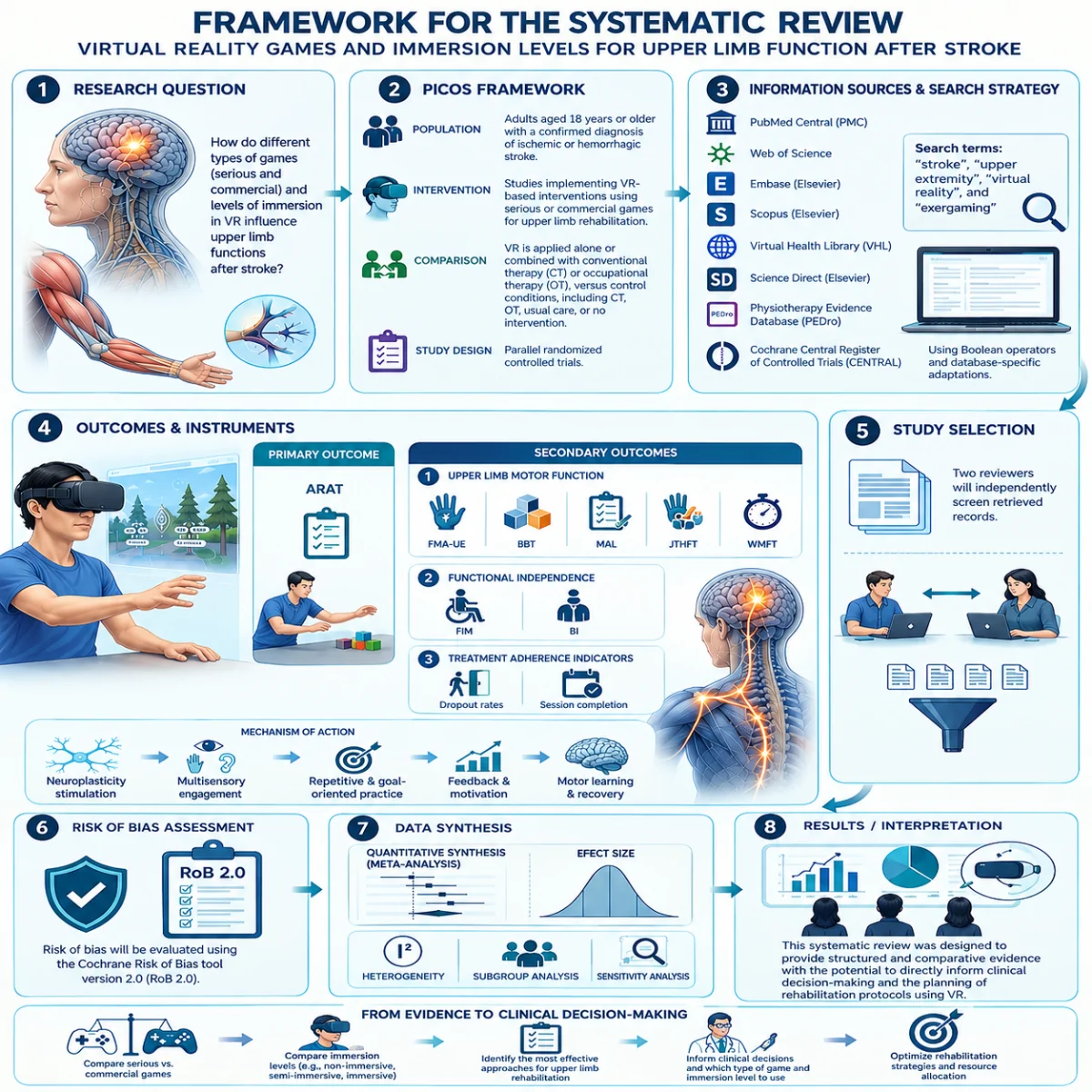
Conceptual and methodological framework of the systematic review. ARAT Action Research Arm Test; BBT Box and Block Test; FMA-UE Fugl-Meyer Assessment-Upper Extremity; JTHFT Jebsen-Taylor Hand Function Test; MAL Motor Activity Log; PICOS participants, interventions, comparisons, outcomes, and study design; RoB risk of bias; VR virtual reality; WMFT Wolf Motor Function Test.

## Results

This systematic review protocol was registered on PROSPERO in 2024 (CRD42024595266). The literature search will include studies published from database inception to February 2026. At the time of manuscript submission, the screening of retrieved records and data extraction procedures are ongoing. Data synthesis and meta-analysis are expected to be completed by November 2026, with publication of the final results anticipated by winter 2026. The study selection process will be presented in a PRISMA flow diagram (supplementary file), reflecting the current status of the review.

## Discussion

### Principal Results

This systematic review is designed to provide structured and comparative evidence with the potential to directly inform clinical decision-making and the planning of rehabilitation protocols using VR. These implications may support therapists in selecting the most appropriate VR interventions according to therapeutic goals, such as prioritizing task-specific training or enhancing patient engagement. By focusing on randomized controlled trials and clearly distinguishing between serious games and commercial games based on predefined criteria, this review may contribute to more informed game selection, considering factors such as cost-effectiveness, task specificity, and therapeutic engagement.

Understanding whether the effectiveness of VR varies according to immersion level or intervention dosage may also support more precise prescription of training parameters, including session duration, frequency, and total exposure. From a research perspective, the findings may further help identify gaps in the current literature, particularly regarding underexplored comparisons between game types and the optimal characteristics of interventions.

### Limitations

Potential limitations include substantial heterogeneity across VR interventions, particularly regarding differences in game design, task specificity, levels of immersion, intervention protocols, and integration with CT, which may limit comparability across studies. Variability in dosage and training intensity, as well as heterogeneity in participant characteristics, may further contribute to inconsistency in outcomes. Additionally, the number of eligible trials and the quality of the available evidence may influence the precision and certainty of the findings. These factors will be carefully considered during data synthesis and interpretation.

### Comparison With Prior Work

Previous systematic reviews have examined the effects of VR interventions in poststroke rehabilitation. However, many of these reviews evaluate VR as a single intervention category and do not differentiate between serious games and commercial games. By explicitly distinguishing these game formats according to predefined criteria, this review aims to contribute to a more detailed understanding of how different types of VR games may influence upper limb rehabilitation outcomes [2,3,13,16-18,21-25].

### Conclusions

This systematic review will synthesize current evidence on the use of serious and commercial VR games for poststroke upper limb rehabilitation. The resulting evidence may support clinical decision-making and guide the selection of VR interventions in neurological physiotherapy practice.

## Appendix

Multimedia Appendix 1 Reproducible search strategies for all electronic databases, including controlled vocabulary, keywords, Boolean operators, and database-specific adaptations used to identify eligible studies.

## Abbreviations

ARAT: Action Research Arm Test
BBT: Box and Block Test
BI: Barthel index
CT: conventional therapy
FIM: Functional Independence Measure
FIVR: fully immersive virtual reality
FMA-UE: Fugl-Meyer Assessment-Upper Extremity
GRADE: Grading of Recommendations Assessment, Development and Evaluation
IMI: Intrinsic Motivation Inventory
JTHFT: Jebsen-Taylor Hand Function Test
MAL: Motor Activity Log
MCID: minimal clinically important difference
NIVR: nonimmersive virtual reality
OT: occupational therapy
PEDro: Physiotherapy Evidence Database
PICOS: participants, interventions, comparisons, outcomes, and study design
PRISMA: Preferred Reporting Items for Systematic Reviews and Meta-Analyses
PRISMA-P: Preferred Reporting Items for Systematic Reviews and Meta-Analyses-Protocols
PROSPERO: International Prospective Register of Systematic Reviews
RoB: risk of bias
SIVR: semi-immersive virtual reality
VR: virtual reality
WHO: World Health Organization
WMFT: Wolf Motor Function Test
