# Supplementary material for: Influence of Immersion Level and Game Type on Virtual Reality Interventions for Upper Limb Function After Stroke: Protocol for a Systematic Review and Meta-Analysis
Source: JMIR Res Protoc. 2026 Oct 6;15:e95135. doi: 10.2196/95135 (PMC13684114; doi:10.2196/95135)
Supplement: Supplementary file 1 [file resprot_v15i1e95135_app1.pdf]

**Influence of Immersion Level and Game Type on Virtual Reality Interventions for Upper Limb Function After Stroke: Protocol for a Systematic Review and Meta-Analysis**

**da Silva et al.**

| DATABASE | SEARCH STRATEGY |
| --- | --- |
| **PUBMED/ MEDLINE** | #1 "Adult"[Mesh] OR "Adults" OR "Adults, Young" OR "Adult, Young" OR "Young Adults" OR "Middle Age" OR "Aged"[Mesh] OR "Elderly" |
| | #2 "Stroke"[Mesh] OR "Strokes" OR "Cerebrovascular Accident" OR "Cerebrovascular Accidents" OR "Cerebral Stroke" OR "Cerebral Strokes" OR "Stroke, Cerebral" OR "Strokes, Cerebral" OR "Cerebrovascular, Apoplexy" OR "Apoplexy, Cerebrovascular" OR "Vascular Accident, Brain" OR "Brain Vascular Accident" OR "Brain Vascular Accidents" OR "Cerebrovascular Stroke" OR "Cerebrovascular Strokes" OR "Stroke, Cerebrovascular" OR "CVA (Cerebrovascular Accident)" OR "Stroke, Acute" OR "Acute Stroke" OR "Acute Strokes" OR "Strokes, Acute" OR "Cerebrovascular Accident, Acute" OR "Acute Cerebrovascular Accident" OR "Acute Cerebrovascular Accidents" OR "Cerebrovascular Accidents, Acute" OR "Hemorrhagic Strokes" OR "Stroke, Hemorrhagic" OR "Intracerebral Hemorrhagic Stroke" OR "Hemorrhagic Stroke, Intracerebral" OR "Intracerebral Hemorrhagic Strokes" OR "Stroke, Intracerebral Hemorrhagic" OR "Intracerebral Hemorrhage Stroke" OR "Hemorrhage Stroke, Intracerebral" OR "Stroke, Intracerebral Hemorrhage" OR "Subarachnoid Hemorrhagic Stroke" OR "Hemorrhagic Stroke, Subarachnoid" OR "Subarachnoid Hemorrhagic Strokes" OR "Ischemic Strokes" OR "Stroke, Ischemic" OR "Ischaemic Stroke" OR "Ischaemic Strokes" OR "Stroke, Ischaemic" OR "Acute Ischemic Stroke" OR "Acute Ischemic Strokes" OR "Ischemic Stroke, Acute" OR "Stroke, Acute Ischemic" OR "Cryptogenic Ischemic Stroke" OR "Cryptogenic Ischemic Strokes" OR "Ischemic Stroke, Cryptogenic" OR "Cryptogenic Embolism Stroke" OR "Cryptogenic Stroke" OR "Cryptogenic Strokes" OR "Stroke, Cryptogenic" OR "Wake-up Stroke" OR "Stroke, Wake-up" OR "Wake up Stroke" OR "Wake-up Strokes" OR “Stroke Rehabilitation”[Mesh] OR “Rehabilitation, Stroke” |
| | #3 "Upper Extremity"[Mesh] OR “Extremities, Upper” OR “Upper Extremities” OR “Extremity, Upper” OR “Upper Limb” OR “Limbs, Upper” OR “Limb, Upper” OR “Upper Limbs” OR “Arms” OR “Upper Arm” OR “Arms, Upper” OR “Arm, Upper” OR “Upper Arms” OR “Brachium” OR “Brachiums” OR “Elbow” OR “Hands” OR “Finger” OR “Thumbs” OR “metacarpos” |
| | #4 ("Virtual Reality"[Mesh] OR "Reality, Virtual" OR "Virtual Reality, Educational" OR "Educational Virtual Realities" OR "Educational Virtual Reality" "Virtual Reality, Instructional" OR "Virtual Reality Exposure Therapy"[Mesh] OR "Virtual Reality Immersion Therapy" OR "Virtual Reality Therapy" OR "Reality Therapy, Virtual" OR "Therapies, Virtual Reality" OR "Therapy, Virtual Reality" OR "Virtual Reality Therapies") OR "Exergaming"[Mesh] OR "Virtual Reality Exercise" OR "Exercises, Virtual Reality" OR "Exercise, Virtual Reality" OR "Virtual Reality Exercises" OR "Active-Video Gaming" OR "Active Video Gaming" OR "Exergames" OR "Exergame" OR "Smart Glasses"[Mesh] OR "Glasses, Smart" OR "Smartglasses" OR "Head Up Display" OR "Displays, Head Up" OR "Head Up Displays" OR "Head Mounted Display" OR "Display, Head Mounted" OR "Displays, Head Mounted" OR "Head Mounted Displays" OR "Mounted Display, Head" OR "Head Worn Display" OR "Head Worn Displays" OR "Google Glass" OR "Glass, Google" OR "Google Glasses" |
| | #5 ((clinical[Title/Abstract] AND trial[Title/Abstract]) OR clinical trials as topic[MeSH Terms] OR clinical trial[Publication Type] OR random*[Title/Abstract] OR random allocation[MeSH Terms] OR therapeutic use[MeSH Subheading]) |

| | |
|---|---|
| | #1 AND #2 AND #3 AND #4 AND #5 |
| | #6 ((("Adult"[Mesh] OR "Adults" OR "Adults, Young" OR "Adult, Young" OR "Young Adults" OR "Middle Age" OR "Aged"[Mesh] OR "Elderly") AND ("Stroke"[Mesh] OR "Strokes" OR "Cerebrovascular Accident" OR "Cerebrovascular Accidents" OR "Cerebral Stroke" OR "Cerebral Strokes" OR "Stroke, Cerebral" OR "Strokes, Cerebral" OR "Cerebrovascular, Apoplexy" OR "Apoplexy, Cerebrovascular" OR "Vascular Accident, Brain" OR "Brain Vascular Accident" OR "Brain Vascular Accidents" OR "Cerebrovascular Stroke" OR "Cerebrovascular Strokes" OR "Stroke, Cerebrovascular" OR "CVA (Cerebrovascular Accident)" OR "Stroke, Acute" OR "Acute Stroke" OR "Acute Strokes" OR "Strokes, Acute" OR "Cerebrovascular Accident, Acute" OR "Acute Cerebrovascular Accident" OR "Acute Cerebrovascular Accidents" OR "Cerebrovascular Accidents, Acute" OR "Hemorrhagic Strokes" OR "Stroke, Hemorrhagic" OR "Intracerebral Hemorrhagic Stroke" OR "Hemorrhagic Stroke, Intracerebral" OR "Intracerebral Hemorrhagic Strokes" OR "Stroke, Intracerebral Hemorrhagic" OR "Intracerebral Hemorrhage Stroke" OR "Hemorrhage Stroke, Intracerebral" OR "Stroke, Intracerebral Hemorrhage" OR "Subarachnoid Hemorrhagic Stroke" OR "Hemorrhagic Stroke, Subarachnoid" OR "Subarachnoid Hemorrhagic Strokes" OR "Ischemic Strokes" OR "Stroke, Ischemic" OR "Ischaemic Stroke" OR "Ischaemic Strokes" OR "Stroke, Ischaemic" OR "Acute Ischemic Stroke" OR "Acute Ischemic Strokes" OR "Ischemic Stroke, Acute" OR "Stroke, Acute Ischemic" OR "Cryptogenic Ischemic Stroke" OR "Cryptogenic Ischemic Strokes" OR "Ischemic Stroke, Cryptogenic" OR "Cryptogenic Embolism Stroke" OR "Cryptogenic Stroke" OR "Cryptogenic Strokes" OR "Stroke, Cryptogenic" OR "Wake-up Stroke" OR "Stroke, Wake-up" OR "Wake up Stroke" OR "Wake-up Strokes" OR "Stroke Rehabilitation"[Mesh] OR "Rehabilitation, Stroke")) AND (("Virtual Reality"[Mesh] OR "Reality, Virtual" OR "Virtual Reality, Educational" OR "Educational Virtual Realities" OR "Educational Virtual Reality" "Virtual Reality, Instructional" OR "Virtual Reality Exposure Therapy"[Mesh] OR "Virtual Reality Immersion Therapy" OR "Virtual Reality Therapy" OR "Reality Therapy, Virtual" OR "Therapies, Virtual Reality" OR "Therapy, Virtual Reality" OR "Virtual Reality Therapies") OR "Exergaming"[Mesh] OR "Virtual Reality Exercise" OR "Exercises, Virtual Reality" OR "Exercise, Virtual Reality" OR "Virtual Reality Exercises" OR "Active-Video Gaming" OR "Active Video Gaming" OR "Exergames" OR "Exergame" OR "Smart Glasses"[Mesh] OR "Glasses, Smart" OR "Smartglasses" OR "Head Up Display" OR "Displays, Head Up" OR "Head Up Displays" OR "Head Mounted Display" OR "Display, Head Mounted" OR "Displays, Head Mounted" OR "Head Mounted Displays" OR "Mounted Display, Head" OR "Head Worn Display" OR "Head Worn Displays" OR "Google Glass" OR "Glass, Google" OR "Google Glasses")) AND (((clinical[Title/Abstract] AND trial[Title/Abstract]) OR clinical trials as topic[MeSH Terms] OR clinical trial[Publication Type] OR random*[Title/Abstract] OR random allocation[MeSH Terms] OR therapeutic use[MeSH Subheading])) |
| **SCIENCE DIRECT** | Title, abstract, keywords: "Upper Extremity" AND "Stroke" AND "Stroke" AND "Virtual Reality" OR "Virtual Reality Exposure Therapy" AND "Adult" NOT "Exoskeleton Device" NOT "Transcranial Direct Current Stimulation" NOT "Gait" |
| **EMBASE (Elservier)** | ('adult'/exp OR 'adult' OR 'adults' OR 'grown-ups' OR 'grownup' OR 'grownups' OR 'aged'/exp OR 'aged' OR 'aged patient' OR 'aged people' OR 'aged person' OR 'aged subject' OR 'elderly' OR 'elderly patient' OR 'elderly people' OR 'elderly person' OR 'elderly subject' OR 'senior citizen' OR 'senium' OR 'stroke patient'/exp OR 'stroke patient' OR 'stroke sufferer' OR 'stroke sufferers' OR 'cerebrovascular accident'/exp OR 'cva' OR 'accident, cerebrovascular' OR 'acute cerebrovascular lesion' OR 'acute focal cerebral vasculopathy' OR 'acute stroke' OR 'apoplectic stroke' OR 'apoplexia' OR 'apoplexy' OR 'blood flow |

| | |
|---|---|
| | disturbance, brain' OR 'brain accident' OR 'brain attack' OR 'brain blood flow disturbance' OR 'brain insult' OR 'brain insultus' OR 'brain vascular accident' OR 'cerebral apoplexia' OR 'cerebral insult' OR 'cerebral stroke' OR 'cerebral vascular accident' OR 'cerebral vascular insufficiency' OR 'cerebro vascular accident' OR 'cerebrovascular accident' OR 'cerebrovascular arrest' OR 'cerebrovascular failure' OR 'cerebrovascular injury' OR 'cerebrovascular insufficiency' OR 'cerebrovascular insult' OR 'cerebrum vascular accident' OR 'cryptogenic stroke' OR 'insultus cerebralis' OR 'ischaemic seizure' OR 'ischemic seizure' OR 'stroke' OR 'thrombotic stroke') NOT ('coronavirus disease 2019'/exp OR '2019 novel coronavirus disease' OR '2019 novel coronavirus epidemic' OR '2019 novel coronavirus infection' OR '2019-ncov disease' OR '2019-ncov infection' OR 'covid' OR 'covid 19' OR 'covid 2019' OR 'covid-10' OR 'covid-19' OR 'covid19' OR 'sars coronavirus 2 infection' OR 'sars-cov-2 disease' OR 'sars-cov-2 infection' OR 'sars-cov2 disease' OR 'sars-cov2 infection' OR 'sarscov2 disease' OR 'sarscov2 infection' OR 'wuhan coronavirus disease' OR 'wuhan coronavirus infection' OR 'coronavirus disease 2' OR 'coronavirus disease 2010' OR 'coronavirus disease 2019' OR 'coronavirus disease-19' OR 'coronavirus infection 2019' OR 'ncov 2019 disease' OR 'ncov 2019 infection' OR 'novel coronavirus 2019 disease' OR 'novel coronavirus 2019 infection' OR 'novel coronavirus disease 2019' OR 'novel coronavirus infection 2019' OR 'paucisymptomatic coronavirus disease 2019' OR 'severe acute respiratory syndrome 2' OR 'severe acute respiratory syndrome cov-2 infection' OR 'severe acute respiratory syndrome coronavirus 2 infection' OR 'severe acute respiratory syndrome coronavirus 2019 infection') NOT ('multiple sclerosis'/exp OR 'ms (multiple sclerosis)' OR 'chariot disease' OR 'disseminated sclerosis' OR 'insular sclerosis' OR 'multiple sclerosis' OR 'sclerosis multiplex' OR 'sclerosis, disseminated' OR 'sclerosis, insular' OR 'sclerosis, multiple') NOT ('transplantation'/exp OR 'graft surgery' OR 'grafting surgery' OR 'serial transplantation' OR 'surgical grafting' OR 'tissue and organ procurement' OR 'transplantation' OR 'transplantation surgery' OR 'transplants') NOT ('cerebral palsy'/exp OR 'brain palsy' OR 'brain paralysis' OR 'central palsy' OR 'central paralysis' OR 'cerebral palsy' OR 'cerebral paralysis' OR 'cerebral paresis' OR 'diplegia spastica' OR 'encephalopathia infantilis' OR 'palsy, cerebral' OR 'spastic diplegia') NOT ('child'/exp OR 'child' OR 'children') AND ('virtual reality'/exp OR 'virtual reality' OR 'virtual reality system'/exp OR 'vr interface' OR 'vr system (virtual reality)' OR 'virtual reality interface' OR 'virtual reality system' OR 'stroke rehabilitation'/exp OR 'ischemic stroke rehabilitation' OR 'post-stroke rehabilitation' OR 'stroke rehab' OR 'stroke rehabilitation' OR 'exergaming'/exp OR 'vr exercise' OR 'vr exergaming' OR 'vr-based exercise' OR 'active video gaming' OR 'active videogaming' OR 'exer-gaming' OR 'exergaming' OR 'virtual reality (vr) -based exercise' OR 'virtual reality (vr) exergaming' OR 'virtual reality exercise' OR 'virtual reality-based exercise' OR 'smart glasses'/exp OR 'ar glasses' OR 'ar head-mounted device' OR 'ar head-mounted display' OR 'ar head-worn display' OR 'ar headset' OR 'ar-hud' OR 'arhmd' OR 'google glass' OR 'google glass-based device' OR 'google glasses' OR 'hmd-ar' OR 'ost-hmd' OR 'augmented reality glasses' OR 'augmented reality head mounted device' OR 'augmented reality head-mounted display' OR 'augmented reality head-up display' OR 'augmented reality head-worn display' OR 'head-mounted display augmented reality' OR 'optical see-through head-mounted display' OR 'see-through hmd' OR 'smart glasses' OR 'smartglasses') AND ('physiotherapy'/exp OR 'physical therapy' OR 'physical therapy (speciality)' OR 'physical therapy (specialty)' OR 'physical therapy modalities' OR 'physical therapy service' OR 'physical therapy speciality' OR 'physical therapy specialty' OR 'physical therapy techniques' OR 'physical treatment' OR 'physio therapy' OR 'physiotherapy' OR 'physiotherapy department' OR 'therapy, |

| | |
|---|---|
| | physical' OR 'occupational therapy'/exp OR 'ergotherapy' OR 'occupation therapy' OR 'occupational therapy' OR 'paediatric occupational therapy' OR 'pediatric occupational therapy' OR 'therapy, occupational' OR 'kinesiotherapy'/exp OR 'sktm (specialized kinesitherapeutic methodology)' OR 'corrective exercise' OR 'exercise movement techniques' OR 'exercise therapy' OR 'exercise treatment' OR 'kinesiotherapeutic intervention' OR 'kinesiotherapeutic method' OR 'kinesiotherapeutic procedure' OR 'kinesiotherapeutic technique' OR 'kinesiotherapeutical treatment' OR 'kinesiotherapy' OR 'kinesitherapeutic exercises' OR 'kinesitherapeutic intervention' OR 'kinesitherapeutic method' OR 'kinesitherapeutic methodology' OR 'kinesitherapeutic procedure' OR 'kinesitherapeutic technique' OR 'kinesitherapeutic treatment' OR 'kinesitherapeutical treatment' OR 'kinesitherapy' OR 'specialised kinesitherapeutic methodology' OR 'specialized kinesitherapeutic methodology' OR 'therapeutic exercise' OR 'therapy, exercise' OR 'treatment, exercise' OR 'exercise'/exp OR 'biometric exercise' OR 'effort' OR 'exercise' OR 'exercise capacity' OR 'exercise performance' OR 'exercise training' OR 'exertion' OR 'fitness training' OR 'fitness workout' OR 'physical conditioning, human' OR 'physical effort' OR 'physical exercise' OR 'physical exertion' OR 'physical work-out' OR 'physical workout') NOT ('mirror therapy'/exp OR 'mvft (mirror visual feedback therapy)' OR 'mirror feedback therapy' OR 'mirror movement therapy' OR 'mirror therapy' OR 'mirror therapy (mt)' OR 'mirror visual feedback' OR 'mirror visual feedback (mvf) therapy' OR 'mirror visual feedback therapy' OR 'mirror visual feedback therapy (mvft)' OR 'mirror visual therapy') NOT ('transcranial magnetic stimulation'/exp OR 'magnetic stimulation, transcranial' OR 'stimulation, transcranial magnetic' OR 'transcranial magnetic stimulation') NOT ('transcranial electrical stimulation'/exp OR 'transcranial electrical stimulation') NOT ('transcranial direct current stimulation'/exp OR 'tdcs (transcranial direct current stimulation)' OR 'transcranial direct current stimulation') NOT ('robotics'/exp OR 'robotics') NOT ('blood flow restriction training'/exp OR 'bfr exercise' OR 'bfr therapy' OR 'bfr training' OR 'blood flow restriction (bfr) exercise' OR 'blood flow restriction (bfr) therapy' OR 'blood flow restriction (bfr) training' OR 'blood flow restriction exercise' OR 'blood flow restriction therapy' OR 'blood flow restriction training') NOT 'pilates'/exp NOT ('acupuncture'/exp OR 'acupuncture' OR 'acupuncture therapy' OR 'shonishin') NOT ('immobilization stress'/exp OR 'immobilisation induced stress' OR 'immobilisation stress' OR 'immobilisation stresses' OR 'immobilization induced stress' OR 'immobilization stress' OR 'immobilization stresses' OR 'restraint induced stress' OR 'restraint stress' OR 'restraint stresses' OR 'stress, immobilisation' OR 'stress, immobilization') NOT ('constraint induced therapy'/exp OR 'constraint induced therapy') AND ('range of motion'/exp OR 'articular rom' OR 'joint rom' OR 'range of motion' OR 'range of motion, articular' OR 'kinematics'/exp OR 'goniometry'/exp OR 'goniometric measurement' OR 'goniometry' OR 'motor performance'/exp OR 'ability, motor' OR 'function, motor' OR 'motor ability' OR 'motor function' OR 'motor performance' OR 'motor skill' OR 'motor skills' OR 'performance, motor' OR 'skill, motor' OR 'cybersickness'/exp OR 'reaching movement'/exp) NOT 'balance'/exp AND ('controlled study'/exp OR 'control group study' OR 'control group trial' OR 'controlled study' OR 'controlled trial' OR 'randomized controlled trial'/exp OR 'controlled trial, randomized' OR 'randomised controlled study' OR 'randomised controlled trial' OR 'randomized controlled study' OR 'randomized controlled trial' OR 'trial, randomized controlled' OR 'controlled clinical trial'/exp OR 'clinical trial, controlled' OR 'controlled clinical comparison' OR 'controlled clinical drug trial' OR 'controlled clinical experiment' OR 'controlled clinical study' OR 'controlled clinical test' OR 'controlled clinical trial') |

| | |
|---|---|
| **BVS (BIREME)** | #1 “Acidente Vascular Cerebral” OR “Accidente Cerebrovascular” OR “Stroke” OR “AVC” OR “AVE” OR “Acidente Cerebral Vascular” OR “Acidente Cerebrovascular” OR “Acidente Vascular Cerebral (AVC)” OR “Acidente Vascular Encefálico” OR “Acidente Vascular do Cérebro” OR “Acidentes Cerebrais Vasculares” OR “Acidentes Cerebrovasculares” OR “Acidentes Vasculares Cerebrais” OR “Apoplexia” OR “Apoplexia Cerebral” OR “Apoplexia Cerebrovascular” OR “Icto Cerebral” OR “Ictus Cerebral” OR “AVC Agudo” OR “Acidente Vascular Cerebral Agudo” |
| | #2 “Extremidade Superior” OR “Extremidad Superior” OR “Upper Extremity” OR “Extremidades Superiores” OR “Membro Superior” OR “Membro Torácico” OR “Membros Superiores” OR “Membros Torácicos” OR “Região da Extremidade Superior” OR “Reabilitação do Acidente Vascular Cerebral” or “Rehabilitación de Accidente Cerebrovascular” OR “Stroke Rehabilitation” |
| | #3 “Realidade Virtual” OR “Realidad Virtual” OR “Virtual Reality” OR “Realidades Virtuais” OR “Realidade Virtual Educativa” OR “Realidade Virtual Instrucional” OR “Realidades Virtuais Educativas” OR “Realidades Virtuais Instrucionais” |
| | #1 AND #2 AND #3 |
| | #4 (“Acidente Vascular Cerebral” OR “Accidente Cerebrovascular” OR “Stroke” OR “AVC” OR “AVE” OR “Acidente Cerebral Vascular” OR “Acidente Cerebrovascular” OR “Acidente Vascular Cerebral (AVC)” OR “Acidente Vascular Encefálico” OR “Acidente Vascular do Cérebro” OR “Acidentes Cerebrais Vasculares” OR “Acidentes Cerebrovasculares” OR “Acidentes Vasculares Cerebrais” OR “Apoplexia” OR “Apoplexia Cerebral” OR “Apoplexia Cerebrovascular” OR “Icto Cerebral” OR “Ictus Cerebral” OR “AVC Agudo” OR “Acidente Vascular Cerebral Agudo” ) AND (“Extremidade Superior” OR “Extremidad Superior” OR “Upper Extremity” OR “Extremidades Superiores” OR “Membro Superior” OR “Membro Torácico” OR “Membros Superiores” OR “Membros Torácicos” OR “Região da Extremidade Superior” OR “Reabilitação do Acidente Vascular Cerebral” or “Rehabilitación de Accidente Cerebrovascular” OR “Stroke Rehabilitation”) AND (“Realidade Virtual” OR “Realidad Virtual” OR “Virtual Reality” OR “Realidades Virtuais” OR “Realidade Virtual Educativa” OR “Realidade Virtual Instrucional” OR “Realidades Virtuais Educativas” OR “Realidades Virtuais Instrucionais” ) |
| **WEB OF SCIENCE** | # 1 ALL=(“Stroke Rehabilitation” OR “Rehabilitation, Stroke” OR “Rehabilitation, Neurological” OR “Neurologic Rehabilitation” OR “Rehabilitation, Neurologic”) |
| | #2 ALL=(“Upper Extremity” OR “Extremities, Upper” OR “Upper Extremities” OR “Extremity, Upper” OR “Membrum superius” OR “Upper Limb” OR “Limbs, Upper” OR “Limb, Upper” OR “Upper Limbs” OR “Arms” OR “Upper Arm” OR “Arms, Upper” OR “Arm, Upper” OR “Upper Arms” OR “Brachium” OR “Brachiums” OR “Elbow” OR “Hands” OR “Finger” OR “Thumbs” OR “metacarpos”) |
| | #3 ALL=(“Virtual Reality” OR “Reality, Virtual” OR “Virtual Reality, Educational” OR “Educational Virtual Realities” OR “Educational Virtual Reality” OR “Reality, Educational Virtual” OR “Virtual Realities, Educational” OR “Virtual Reality, Instructional” OR “Instructional Virtual Realities” OR “Instructional Virtual Reality” OR “Realities, Instructional Virtual” OR “Reality, Instructional Virtual” OR “Virtual Realities, Instructional”) |
| | #4 ALL =(#1 AND #2 AND #3) |
| | ((TS=((“Stroke Rehabilitation” OR “Rehabilitation, Stroke” OR “Rehabilitation, Neurological” OR “Neurologic Rehabilitation” OR “Rehabilitation, Neurologic”))) AND TS=((“Upper Extremity” OR “Extremities, Upper” OR “Upper Extremities” OR “Extremity, Upper” OR “Membrum superius” OR “Upper Limb” OR “Limbs, |

| | |
|---|---|
| | Upper” OR “Limb, Upper” OR “Upper Limbs” OR “Arms” OR “Upper Arm” OR “Arms, Upper” OR “Arm, Upper” OR “Upper Arms” OR “Brachium” OR “Brachiums” OR “Elbow” OR “Hands” OR “Finger” OR “Thumbs” OR “metacarpos”))) AND TS=((“Virtual Reality” OR “Reality, Virtual” OR “Virtual Reality, Educational” OR “Educational Virtual Realities” OR “Educational Virtual Reality” OR “Reality, Educational Virtual” OR “Virtual Realities, Educational” OR “Virtual Reality, Instructional” OR “Instructional Virtual Realities” OR “Instructional Virtual Reality” OR “Realities, Instructional Virtual” OR “Reality, Instructional Virtual” OR “Virtual Realities, Instructional”)) |
| **SCOPUS (Elsevier)** | #1 TITLE-ABS-KEY("stroke rehabilitation" OR "rehabilitation, stroke" OR "neurological rehabilitation" OR "rehabilitation, neurological" OR "neurological rehabilitation" OR "adult" OR "adults" OR "adults, young" OR "adult, young" OR "young adults" OR "middle age" OR "Aged" OR "Elderly") |
| | #2 ( "upper extremity" OR "upper extremities" OR "upper extremity" OR " membrum superius" OR "upper limb" OR "upper limbs" OR "upper limb" OR "upper limbs" OR "arms" OR "upper arm" OR "upper arms" OR "upper arm" OR "upper arms" OR "upper arms" OR "upper arm" OR "upper arms" OR "arm" OR "arms" OR "arms" OR "elbow" OR "hands" OR "finger" OR "thumbs" OR "metacarpals" OR "biomechanical phenomena" OR "phenomena, biomechanical" OR "biomechanic phenomena" OR "biomechanic phenomenas" OR "phenomena, biomechanic" OR "phenomenas, biomechanic" OR "biomechanics" OR "biomechanic" OR "kinematics" ) |
| | #3 TITLE-ABS-KEY ( "reality, virtual" OR "virtual reality, educational" OR "educational virtual realities" OR "educational virtual reality" OR "reality, educational virtual" OR "virtual realities, educational" OR "virtual reality, instructional" OR "instructional virtual realities" OR "instructional virtual reality" OR "realities, instructional virtual" OR "reality, instructional virtual" OR "virtual realities, instructional" OR "virtual reality exposure therapy" OR "virtual reality immersion therapy" OR "virtual reality therapy" OR "reality therapies, virtual" OR "reality therapy, virtual" OR "therapies, virtual reality" OR "therapy, virtual reality" OR "virtual reality therapies" ) |
| | #4 TITLE-ABS-KEY ( "covid-19" OR "2019-ncov infection" OR "2019 ncov infection" OR "2019-ncov infections" OR "infection, 2019-ncov" OR "sars-cov-2 infection" OR "infection, sars-cov-2" OR "sars cov 2 infection" OR "sars-cov-2 infections" OR "2019 novel coronavirus disease" OR "2019 novel coronavirus infection" OR "2019-ncov disease" OR "2019 ncov disease" OR "2019-ncov diseases" OR "disease, 2019-ncov" OR "covid19" OR "coronavirus disease 2019" OR "disease 2019, coronavirus" OR "coronavirus disease-19" OR "coronavirus disease 19" OR "severe acute respiratory syndrome coronavirus 2 infection" OR "covid-19 virus disease" OR "covid 19 virus disease" OR "covid-19 virus diseases" OR "disease, covid-19 virus" OR "virus disease, covid-19" OR "sars coronavirus 2 infection" OR "covid-19 virus infection" OR "covid 19 virus infection" OR "covid-19 virus infections" OR "infection, covid-19 virus" OR "virus infection, covid-19" OR "covid-19 pandemic" OR "covid 19 pandemic" OR "pandemic, covid-19" OR "covid-19 pandemics" ) |
| | #5 TITLE-ABS-KEY ( "transcranial direct current stimulation" OR "tdcs" OR "anodal stimulation" OR "transcranial direct current stimulation" OR "anodal stimulation tdcs" OR "anodal stimulation tdcss" OR "stimulation tdcs, anodal" OR "stimulation tdcss, anodal" OR "tdcs, anodal stimulation" OR "tdcss, anodal stimulation" OR "cathodal stimulation transcranial direct current stimulation" OR "cathodal stimulation tdcs" OR "cathodal stimulation tdcss" OR "stimulation tdcs, cathodal" OR "stimulation tdcss, cathodal" OR "tdcs, cathodal stimulation" OR "tdcss, cathodal stimulation" OR "transcranial alternating current stimulation" OR "transcranial random noise stimulation" OR "repetitive |

| | |
|---|---|
| | transcranial electrical stimulation" OR "transcranial electrical stimulation" OR "electrical stimulations, transcranial" OR "electrical stimulation, transcranial" OR "stimulations, transcranial electrical" OR "stimulation, transcranial electrical" ) |
| | #6 TITLE-ABS-KEY ( "magnetic stimulations, transcranial" OR "magnetic stimulation, transcranial" OR "stimulations, transcranial magnetic" OR "stimulation, transcranial magnetic" OR "transcranial magnetic stimulations" OR "transcranial magnetic stimulation, paired pulse" OR "transcranial magnetic stimulation, repetitive" OR "transcranial magnetic stimulation, single pulse" ) |
| | #7 TITLE-ABS-KEY ( "robotics" OR "remote operations (robotics)" OR "operation, remote (robotics)" OR "operations, remote (robotics)" OR "remote operation (robotics)" OR "telerobotics" OR "soft robotics" OR "robotic, soft" OR "soft robotic" OR "socially assistive robots" OR "assistive robot, socially" OR "robot, socially assistive" OR "socially assistive robot" OR "social robots" OR "robot, social" OR "social robot" OR "humanoid robots" OR "humanoid robot" OR "robot, humanoid" OR "companion robots" OR "companion robot" OR "robot, companion" ) |
| | #8 TITLE-ABS-KEY ( "gait" OR "gaits" OR "postural balance" OR "posture balance" OR "balance, posture" OR "posture balances" OR "equilibrium, posture" OR "musculoskeletal equilibrium" OR "equilibrium, musculoskeletal" OR "postural equilibrium" OR "equilibrium, postural" OR "balance, postural" OR "postural control" OR "control, postural" OR "postural controls" OR "posture control" OR "control, posture" OR "posture controls" ) |
| | #9 TITLE-ABS-KEY ( "mirror movement therapy" OR "mirror movement therapies" OR "movement therapies, mirror" OR "movement therapy, mirror" OR "therapies, mirror movement" OR "therapy, mirror movement" OR "mirror therapy" ) |
| | #1 AND #2 AND #3 NOT #4 NOT #5 NOT #6 NOT #7 NOT #8 NOT #9 |
| | #10 ( TITLE-ABS-KEY ( "stroke rehabilitation" OR "rehabilitation, stroke" OR "neurological rehabilitation" OR "rehabilitation, neurological" OR "neurological rehabilitation" OR "adult" OR "adults" OR "adults, young" OR "adult, young" OR "young adults" OR "middle age" OR "aged" OR "elderly" ) AND TITLE-ABS-KEY ( "upper extremity" OR "upper extremities" OR "upper extremity" OR " membrum superius" OR "upper limb" OR "upper limbs" OR "upper limb" OR "upper limbs" OR "arms" OR "upper arm" OR "upper arms" OR "upper arm" OR "upper arms" OR "upper arms" OR "upper arm" OR "upper arms" OR "arm" OR "arms" OR "arms" OR "elbow" OR "hands" OR "finger" OR "thumbs" OR "metacarpals" OR "biomechanical phenomena" OR "phenomena, biomechanical" OR "biomechanic phenomena" OR "biomechanic phenomenas" OR "phenomena, biomechanic" OR "phenomenas, biomechanic" OR "biomechanics" OR "biomechanic" OR "kinematics" ) AND TITLE-ABS-KEY ( "reality, virtual" OR "virtual reality, educational" OR "educational virtual realities" OR "educational virtual reality" OR "reality, educational virtual" OR "virtual realities, educational" OR "virtual reality, instructional" OR "instructional virtual realities" OR "instructional virtual reality" OR "realities, instructional virtual" OR "reality, instructional virtual" OR "virtual realities, instructional" OR "virtual reality exposure therapy" OR "virtual reality immersion therapy" OR "virtual reality therapy" OR "reality therapies, virtual" OR "reality therapy, virtual" OR "therapies, virtual reality" OR "therapy, virtual reality" OR "virtual reality therapies" ) AND NOT TITLE-ABS-KEY ( "covid-19" OR "2019-ncov infection" OR "2019 ncov infection" OR "2019-ncov infections" OR "infection, 2019-ncov" OR "sars-cov-2 infection" OR "infection, sars-cov-2" OR "sars cov 2 infection" OR "sars-cov-2 infections" OR "2019 novel coronavirus disease" OR "2019 novel coronavirus infection" OR "2019-ncov disease" OR "2019 ncov disease" OR "2019-ncov diseases" OR "disease, 2019-ncov" OR "covid19" OR "coronavirus disease 2019" OR "disease 2019, coronavirus" OR "coronavirus |

| | |
|---|---|
| | disease-19" OR "coronavirus disease 19" OR "severe acute respiratory syndrome coronavirus 2 infection" OR "covid-19 virus disease" OR "covid 19 virus disease" OR "covid-19 virus diseases" OR "disease, covid-19 virus" OR "virus disease, covid-19" OR "sars coronavirus 2 infection" OR "covid-19 virus infection" OR "covid 19 virus infection" OR "covid-19 virus infections" OR "infection, covid-19 virus" OR "virus infection, covid-19" OR "covid-19 pandemic" OR "covid 19 pandemic" OR "pandemic, covid-19" OR "covid-19 pandemics" ) AND NOT TITLE-ABS-KEY ( "transcranial direct current stimulation" OR "tdcs" OR "anodal stimulation" OR "transcranial direct current stimulation" OR "anodal stimulation tdcs" OR "anodal stimulation tdcss" OR "stimulation tdcs, anodal" OR "stimulation tdcss, anodal" OR "tdcs, anodal stimulation" OR "tdcss, anodal stimulation" OR "cathodal stimulation transcranial direct current stimulation" OR "cathodal stimulation tdcs" OR "cathodal stimulation tdcss" OR "stimulation tdcs, cathodal" OR "stimulation tdcss, cathodal" OR "tdcs, cathodal stimulation" OR "tdcss, cathodal stimulation" OR "transcranial alternating current stimulation" OR "transcranial random noise stimulation" OR "repetitive transcranial electrical stimulation" OR "transcranial electrical stimulation" OR "electrical stimulations, transcranial" OR "electrical stimulation, transcranial" OR "stimulations, transcranial electrical" OR "stimulation, transcranial electrical" ) AND NOT TITLE-ABS-KEY ( "magnetic stimulations, transcranial" OR "magnetic stimulation, transcranial" OR "stimulations, transcranial magnetic" OR "stimulation, transcranial magnetic" OR "transcranial magnetic stimulations" OR "transcranial magnetic stimulation, paired pulse" OR "transcranial magnetic stimulation, repetitive" OR "transcranial magnetic stimulation, single pulse" ) AND NOT TITLE-ABS-KEY ( "robotics" OR "remote operations (robotics)" OR "operation, remote (robotics)" OR "operations, remote (robotics)" OR "remote operation (robotics)" OR "telerobotics" OR "soft robotics" OR "robotic, soft" OR "soft robotic" OR "socially assistive robots" OR "assistive robot, socially" OR "robot, socially assistive" OR "socially assistive robot" OR "social robots" OR "robot, social" OR "social robot" OR "humanoid robots" OR "humanoid robot" OR "robot, humanoid" OR "companion robots" OR "companion robot" OR "robot, companion" ) AND NOT TITLE-ABS-KEY ( "gait" OR "gaits" OR "postural balance" OR "posture balance" OR "balance, posture" OR "posture balances" OR "equilibrium, posture" OR "musculoskeletal equilibrium" OR "equilibrium, musculoskeletal" OR "postural equilibrium" OR "equilibrium, postural" OR "balance, postural" OR "postural control" OR "control, postural" OR "postural controls" OR "posture control" OR "control, posture" OR "posture controls" ) AND NOT TITLE-ABS-KEY ( "mirror movement therapy" OR "mirror movement therapies" OR "movement therapies, mirror" OR "movement therapy, mirror" OR "therapies, mirror movement" OR "therapy, mirror movement" OR "mirror therapy" ) ) |
| **CINAHL (EBSCO)** | #1 (MH "Adult") OR (MH "Aged") OR (MH "Aged, 80 and Over") OR (MH "Stroke") OR (MH "Ischemic Stroke") OR (MH "Hemorrhagic Stroke") OR (MH "Stroke Patients") OR (MH "Cerebral Infarction") OR (MH "Embolic Stroke") OR (MH "Stroke, Lacunar") |
| | #2 (MH "Upper Extremity") OR (MH "Upper Extremity Exercises") OR (MH "Forearm") OR (MH "Elbow") OR (MH "Axilla") OR (MH "Arm") OR (MH "Hand") OR (MH "Fingers") OR (MH "Wrist") OR (MH "Thumb") OR (MH "Rehabilitation") OR (MH "Arm Exercises") OR (MH "Therapeutic Exercise") |
| | #3 (MH "Virtual Reality") OR (MH "Virtual Reality Exposure Therapy") OR (MH "Reality Therapy") OR (MH "Video Games") OR (MH "Exergames") OR (MH "Immersion") OR (MH "Smart Glasses") |
| | #4 (MH "Range of Motion") OR (MH "Range of Motion (Saba CCC)") OR (MH "Movement") OR (MH "Adduction") OR (MH "Extension") OR (MH "Hyperextension") OR (MH "Flexion") OR (MH "Reaching") OR (MH |

| | |
|---|---|
| | "Kinematics") OR (MH "Motivation") OR (MH "Kinesiotherapy") OR (MH "Occupational Therapy") OR (MH "Physical Therapy") OR (MH "Abduction") |
| | #5 (MH "Acupuncture") OR (MH "Acupuncture Analgesia") OR (MH "Acupuncture Anesthesia") OR (MH "Electroacupuncture") OR (MH "Chiropractic") OR (MH "Homeopathy") OR (MH "Integrative Medicine") OR (MH "Medicine, Environmental") OR (MH "Robotics") OR (MH "Exoskeleton Devices") OR (MH "Parkinson Disease") OR (MH "Parkinsonian Disorders") OR (MH "Kufor-Rakeb Syndrome") OR (MH "Lewy Body Disease") OR (MH "Transcutaneous Electric Nerve Stimulation") OR (MH "Transcutaneous Electrical Nerve Stimulation (Iowa NIC)") OR (MH "Gait") OR (MH "Walking") OR (MH "Step") OR (MH "Walking Speed") OR (MH "Balance, Postural") OR (MH "Balance Training, Physical") OR (MH "Balance (Iowa NOC)") OR (MH "Exercise Therapy: Balance (Iowa NIC)") OR (MH "Constraint-Induced Therapy") OR (MH "Blood Flow Restriction Training") OR (MH "Cryotherapy") OR (MH "Electrotherapy") OR (MH "Gait Training") OR (MH "Mirror Therapy") OR (MH "Pediatric Physical Therapy") |
| | #1 AND #2 AND #3 AND #4 NOT #5 |
| | #5 ( (MH "Adult") OR (MH "Aged") OR (MH "Aged, 80 and Over") OR (MH "Stroke") OR (MH "Ischemic Stroke") OR (MH "Hemorrhagic Stroke") OR (MH "Stroke Patients") OR (MH "Cerebral Infarction") OR (MH "Embolic Stroke") OR (MH "Stroke, Lacunar") ) AND ( (MH "Upper Extremity") OR (MH "Upper Extremity Exercises") OR (MH "Forearm") OR (MH "Elbow") OR (MH "Axilla") OR (MH "Arm") OR (MH "Hand") OR (MH "Fingers") OR (MH "Wrist") OR (MH "Thumb") OR (MH "Rehabilitation") OR (MH "Arm Exercises") OR (MH "Therapeutic Exercise") ) AND ( (MH "Virtual Reality") OR (MH "Virtual Reality Exposure Therapy") OR (MH "Reality Therapy") OR (MH "Video Games") OR (MH "Exergames") OR (MH "Immersion") OR (MH "Smart Glasses") ) AND ( (MH "Range of Motion") OR (MH "Range of Motion (Saba CCC)") OR (MH "Movement") OR (MH "Adduction") OR (MH "Extension") OR (MH "Hyperextension") OR (MH "Flexion") OR (MH "Reaching") OR (MH "Kinematics") OR (MH "Motivation") OR (MH "Kinesiotherapy") OR (MH "Occupational Therapy") OR (MH "Physical Therapy") OR (MH "Abduction") ) NOT ( (MH "Acupuncture") OR (MH "Acupuncture Analgesia") OR (MH "Acupuncture Anesthesia") OR (MH "Electroacupuncture") OR (MH "Chiropractic") OR (MH "Homeopathy") OR (MH "Integrative Medicine") OR (MH "Medicine, Environmental") OR (MH "Robotics") OR (MH "Exoskeleton Devices") OR (MH "Parkinson Disease") OR (MH "Parkinsonian Disorders") OR (MH "Kufor-Rakeb Syndrome") OR (MH "Lewy Body Disease") OR (MH "Transcutaneous Electric Nerve Stimulation") OR (MH "Transcutaneous Electrical Nerve Stimulation (Iowa NIC)") OR (MH "Gait") OR (MH "Walking") OR (MH "Step") OR (MH "Walking Speed") OR (MH "Balance, Postural") OR (MH "Balance Training, Physical") OR (MH "Balance (Iowa NOC)") OR (MH "Exercise Therapy: Balance (Iowa NIC)") OR (MH "Constraint-Induced Therapy") OR (MH "Blood Flow Restriction Training") OR (MH "Cryotherapy") OR (MH "Electrotherapy") OR (MH "Gait Training") OR (MH "Mirror Therapy") OR (MH "Pediatric Physical Therapy") ) |
| **PEDRO** | Stroke Rehabilitation Virtual reality upper extremity |
| **CENTRAL** | #1 "Adult" OR “Adults” OR “Adults, Young” OR “Adult, Young” OR “Young Adults” OR “Middle Age” OR "Aged" OR "Elderly" OR “Stroke Patients” |
| | #2 "Stroke" OR "Strokes" OR "Cerebrovascular Accident" OR "Cerebrovascular Accidents" OR "Cerebral Stroke" OR "Cerebral Strokes" OR "Stroke, Cerebral" OR "Strokes, Cerebral" OR "Cerebrovascular, Apoplexy" OR "Apoplexy, Cerebrovascular" OR "Vascular Accident, Brain" OR "Brain Vascular Accident" OR "Brain Vascular Accidents" OR "Cerebrovascular Stroke" OR "Cerebrovascular Strokes" OR "Stroke, Cerebrovascular" OR "CVA |

| | |
|---|---|
| | (Cerebrovascular Accident)" OR "Stroke, Acute" OR "Acute Stroke" OR "Acute Strokes" OR "Strokes, Acute" OR "Cerebrovascular Accident, Acute" OR "Acute Cerebrovascular Accident" OR "Acute Cerebrovascular Accidents" OR "Cerebrovascular Accidents, Acute" OR "Hemorrhagic Strokes" OR "Stroke, Hemorrhagic" OR "Intracerebral Hemorrhagic Stroke" OR "Hemorrhagic Stroke, Intracerebral" OR "Intracerebral Hemorrhagic Strokes" OR "Stroke, Intracerebral Hemorrhagic" OR "Intracerebral Hemorrhage Stroke" OR "Hemorrhage Stroke, Intracerebral" OR "Stroke, Intracerebral Hemorrhage" OR "Subarachnoid Hemorrhagic Stroke" OR "Hemorrhagic Stroke, Subarachnoid" OR "Subarachnoid Hemorrhagic Strokes" OR "Ischemic Strokes" OR "Stroke, Ischemic" OR "Ischaemic Stroke" OR "Ischaemic Strokes" OR "Stroke, Ischaemic" OR "Acute Ischemic Stroke" OR "Acute Ischemic Strokes" OR "Ischemic Stroke, Acute" OR "Stroke, Acute Ischemic" OR "Cryptogenic Ischemic Stroke" OR "Cryptogenic Ischemic Strokes" OR "Ischemic Stroke, Cryptogenic" OR "Cryptogenic Embolism Stroke" OR "Cryptogenic Stroke" OR "Cryptogenic Strokes" OR "Stroke, Cryptogenic" OR "Wake-up Stroke" OR "Stroke, Wake-up" OR "Wake up Stroke" OR "Wake-up Strokes" |
| | #3 "Upper Extremity" OR “Extremities, Upper” OR “Upper Extremities” OR “Extremity, Upper” OR “Upper Limb” OR “Limbs, Upper” OR “Limb, Upper” OR “Upper Limbs” OR “Arms” OR “Upper Arm” OR “Arms, Upper” OR “Arm, Upper” OR “Upper Arms” OR “Brachium” OR “Brachiums” OR “Elbow” OR “Hands” OR “Finger” OR “Thumbs” OR “metacarpos” |
| | #4 "Virtual Reality" OR "Reality, Virtual" OR "Virtual Reality, Educational" OR "Educational Virtual Realities" OR "Educational Virtual Reality" "Virtual Reality, Instructional" OR "Virtual Reality Exposure Therapy" OR "Virtual Reality Immersion Therapy" OR "Virtual Reality Therapy" OR "Reality Therapy, Virtual" OR "Therapies, Virtual Reality" OR "Therapy, Virtual Reality" OR "Virtual Reality Therapies" OR "Exergaming" OR "Virtual Reality Exercise" OR "Exercises, Virtual Reality" OR "Exercise, Virtual Reality" OR "Virtual Reality Exercises" OR "Active-Video Gaming" OR "Active Video Gaming" OR "Exergames" OR "Exergame" OR "Smart Glasses" OR "Glasses, Smart" OR "Smartglasses" OR "Head Up Display" OR "Displays, Head Up" OR "Head Up Displays" OR "Head Mounted Display" OR "Display, Head Mounted" OR "Displays, Head Mounted" OR "Head Mounted Displays" OR "Mounted Display, Head" OR "Head Worn Display" OR "Head Worn Displays" OR "Google Glass" OR "Glass, Google" OR "Google Glasses" |
| | #1 AND #2 AND #3 AND #4 |
| | #5 - ("Adult" OR “Adults” OR “Adults, Young” OR “Adult, Young” OR “Young Adults” OR “Middle Age” OR "Aged" OR "Elderly" OR “Stroke Patients”):ti,ab,kw AND ("Stroke" OR "Strokes" OR "Cerebrovascular Accident" OR "Cerebrovascular Accidents" OR "Cerebral Stroke" OR "Cerebral Strokes" OR "Stroke, Cerebral" OR "Strokes, Cerebral" OR "Cerebrovascular, Apoplexy" OR "Apoplexy, Cerebrovascular" OR "Vascular Accident, Brain" OR "Brain Vascular Accident" OR "Brain Vascular Accidents" OR "Cerebrovascular Stroke" OR "Cerebrovascular Strokes" OR "Stroke, Cerebrovascular" OR "CVA (Cerebrovascular Accident)" OR "Stroke, Acute" OR "Acute Stroke" OR "Acute Strokes" OR "Strokes, Acute" OR "Cerebrovascular Accident, Acute" OR "Acute Cerebrovascular Accident" OR "Acute Cerebrovascular Accidents" OR "Cerebrovascular Accidents, Acute" OR "Hemorrhagic Strokes" OR "Stroke, Hemorrhagic" OR "Intracerebral Hemorrhagic Stroke" OR "Hemorrhagic Stroke, Intracerebral" OR "Intracerebral Hemorrhagic Strokes" OR "Stroke, Intracerebral Hemorrhagic" OR "Intracerebral Hemorrhage Stroke" OR "Hemorrhage Stroke, Intracerebral" OR "Stroke, Intracerebral Hemorrhage" OR "Subarachnoid Hemorrhagic Stroke" OR "Hemorrhagic Stroke, Subarachnoid" OR "Subarachnoid Hemorrhagic Strokes" OR "Ischemic Strokes" OR "Stroke, |

| | |
|---|---|
| | Ischemic" OR "Ischaemic Stroke" OR "Ischaemic Strokes" OR "Stroke, Ischaemic" OR "Acute Ischemic Stroke" OR "Acute Ischemic Strokes" OR "Ischemic Stroke, Acute" OR "Stroke, Acute Ischemic" OR "Cryptogenic Ischemic Stroke" OR "Cryptogenic Ischemic Strokes" OR "Ischemic Stroke, Cryptogenic" OR "Cryptogenic Embolism Stroke" OR "Cryptogenic Stroke" OR "Cryptogenic Strokes" OR "Stroke, Cryptogenic" OR "Wake-up Stroke" OR "Stroke, Wake-up" OR "Wake up Stroke" OR "Wake-up Strokes"):ti,ab,kw AND ("Upper Extremity" OR “Extremities, Upper” OR “Upper Extremities” OR “Extremity, Upper” OR “Upper Limb” OR “Limbs, Upper” OR “Limb, Upper” OR “Upper Limbs” OR “Arms” OR “Upper Arm” OR “Arms, Upper” OR “Arm, Upper” OR “Upper Arms” OR “Brachium” OR “Brachiums” OR “Elbow” OR “Hands” OR “Finger” OR “Thumbs” OR “metacarpos”):ti,ab,kw AND ("Virtual Reality" OR "Reality, Virtual" OR "Virtual Reality, Educational" OR "Educational Virtual Realities" OR "Educational Virtual Reality" "Virtual Reality, Instructional" OR "Virtual Reality Exposure Therapy" OR "Virtual Reality Immersion Therapy" OR "Virtual Reality Therapy" OR "Reality Therapy, Virtual" OR "Therapies, Virtual Reality" OR "Therapy, Virtual Reality" OR "Virtual Reality Therapies" OR "Exergaming" OR "Virtual Reality Exercise" OR "Exercises, Virtual Reality" OR "Exercise, Virtual Reality" OR "Virtual Reality Exercises" OR "Active-Video Gaming" OR "Active Video Gaming" OR "Exergames" OR "Exergame" OR "Smart Glasses" OR "Glasses, Smart" OR "Smartglasses" OR "Head Up Display" OR "Displays, Head Up" OR "Head Up Displays" OR "Head Mounted Display" OR "Display, Head Mounted" OR "Displays, Head Mounted" OR "Head Mounted Displays" OR "Mounted Display, Head" OR "Head Worn Display" OR "Head Worn Displays" OR "Google Glass" OR "Glass, Google" OR "Google Glasses"):ti,ab,kw" (Word variations have been searched) |
| **TOTAL** | |

**Legend:** PubMed, Web of Science, Embase (Elsevier), Scopus (Elsevier), Virtual Health Library (BVS), Science Direct (Elsevier), Physiotherapy Evidence Database (PEDro), and CENTRAL.
